# Automated Detection of High-Frequency Oscillations in Epilepsy Based on a Convolutional Neural Network

**DOI:** 10.3389/fncom.2019.00006

**Published:** 2019-02-12

**Authors:** Rui Zuo, Jing Wei, Xiaonan Li, Chunlin Li, Cui Zhao, Zhaohui Ren, Ying Liang, Xinling Geng, Chenxi Jiang, Xiaofeng Yang, Xu Zhang

**Affiliations:** ^1^School of Biomedical Engineering, Capital Medical University, Beijing, China; ^2^Beijing Key Laboratory of Fundamental Research on Biomechanics in Clinical Application, Capital Medical University, Beijing, China; ^3^Neuroelectrophysiological Laboratory, Xuanwu Hospital, Capital Medical University, Beijing, China; ^4^Center of Epilepsy, Beijing Institute for Brain Disorders, Beijing, China

**Keywords:** epilepsy, convolutional neural network, high-frequency oscillations, ripples, fast ripples, automated detection

## Abstract

Epilepsy is one of the most common chronic neurological diseases. High-frequency oscillations (HFOs) have emerged as promising biomarkers for the epileptogenic zone. However, visual marking of HFOs is a time-consuming and laborious process. Several automated techniques have been proposed to detect HFOs, yet these are still far from being suitable for application in a clinical setting. Here, ripples and fast ripples from intracranial electroencephalograms were detected in six patients with intractable epilepsy using a convolutional neural network (CNN) method. This approach proved more accurate than using four other HFO detectors integrated in RIPPLELAB, providing a higher sensitivity (77.04% for ripples and 83.23% for fast ripples) and specificity (72.27% for ripples and 79.36% for fast ripples) for HFO detection. Furthermore, for one patient, the Cohen's kappa coefficients comparing automated detection and visual analysis results were 0.541 for ripples and 0.777 for fast ripples. Hence, our automated detector was capable of reliable estimates of ripples and fast ripples with higher sensitivity and specificity than four other HFO detectors. Our detector may be used to assist clinicians in locating epileptogenic zone in the future.

## Introduction

Epilepsy is one of the most common chronic neurological diseases, with an incidence of between 0.5 and 1% (Jacobs et al., 2012; Chaibi et al., 2013), affecting about 67 million people worldwide (Holden et al., 2005; Makeyev et al., 2017). Most patients are treated successfully with antiepileptic drugs, although about 30% still suffer from medically refractory epilepsy (Kwan and Brodie, 2000; Pati and Alexopoulos, 2010; Tamilia et al., 2017). For these individuals, surgical removal of the epileptogenic zone (EZ), where such seizures originate, is considered the most promising treatment; however, surgical resection depends on correct delimitation of the EZ (Jacobs et al., 2012; Tamilia et al., 2017). Accurate delimitation of the EZ is the main determinant of successful epilepsy surgery.

High-frequency oscillations (HFOs) have been defined as events with four consecutive oscillations between 80 and 500 Hz that clearly rise above the baseline (Zelmann et al., 2009). Another definition is a root mean square (RMS) amplitude increase of more than five times the standard deviation compared with background electroencephalogram (EEG), a duration of at least 6 ms, and more than six peaks (positive plus negative) more than three standard deviations above the mean baseline (Staba et al., 2002). Traditionally, EEG frequencies are believed to be relevant up to the beta, theta, and gamma band (Wang et al., 2017; Yan et al., 2017a,b). But recent findings in rodents and humans have shown a possible relation between HFOs and the EZ (Bragin et al., 1999; Staba et al., 2002; Jacobs et al., 2010). Furthermore, two post-surgical studies have indicated a good correlation between surgical outcome and the removal of tissue corresponding to channels with high HFO rates (Jacobs et al., 2010; Wu et al., 2010). HFOs have gradually emerged as promising new biomarkers for the identification of EZ (Jirsch et al., 2006; Jacobs et al., 2010, 2012; Chou et al., 2016; Cimbalnik et al., 2016; Fedele et al., 2016). HFOs can be subdivided according to their spectral range into ripples (80–200 Hz) and fast ripples (200–500 Hz, FRs) (Jacobs et al., 2012; Pail et al., 2013). Whereas, ripples may reflect inhibitory field potentials that synchronize neuronal activity, thus facilitating information transfer over long distances, fast ripples are pathological and are believed to reflect summated action potentials of spontaneously bursting neurons (Cendes and Meador, 2018).

Nevertheless, detection of HFOs is complicated and time-consuming owing to their short duration and low amplitude (Lopez-Cuevas et al., 2013; Gliske et al., 2016). Existing detection methods can be categorized into automated detection and visual marking, which is a highly time-consuming process (it takes about 10 h to visually mark HFOs in a ten-channel 10-min recording) (Staba et al., 2002; Gardner et al., 2007; Zelmann et al., 2009), and prone to reviewer bias and drift in judgement (Cimbalnik et al., 2018). As a consequence, the development of automated HFO detectors is crucial for the eventual utilization of HFOs in clinical settings.

Several automated HFO detectors have been developed by different research groups. In 2002, Staba et al. (2002) introduced automated detection of HFOs based on the RMS feature of the band-pass-filtered signals. Thresholding-based approaches have become popular since the pioneering work of Staba et al. (2002), for example, those based on short-time line-length (Gardner et al., 2007), complex Morlet wavelet transforms (Chaibi et al., 2013), the Hilbert envelope (Dumpelmann et al., 2012), and approximate entropy (Lopez-Cuevas et al., 2013). Since 2010, detection algorithms have been designed to tackle the problem of low specificity through various approaches. Dumpelmann et al. (2012) chose signal power, line-length, and instantaneous frequency as input features, and used a radial basis function neural network to detect HFOs. Zelmann et al. (2010) improved the RMS detector by computing the energy threshold from baseline segments, Chaibi et al. (2013) combined RMS and empiric mode decomposition, and Ren et al. (2018) used the maximum distributed peak points method to improve baseline determination accuracy. However, most of the automated HFO processing methods still had drawbacks such as low specificity and high rates of false positives. These detectors are still unsuitable for application in a clinical setting.

In recent years, deep learning has been widely applied in diverse domains such as computer vision, natural language processing, and speech recognition (LeCun et al., 2015). It forms the basis of various machine learning algorithms that model high-level data abstractions, and does not rely on handcrafted features (LeCun et al., 2015; Schmidhuber, 2015). The convolutional neural network (CNN), as a deep learning algorithm, has shown remarkable performance in challenging two-dimensional (2D) medical image computing problems, such as classification of lung image patches with interstitial lung disease (Li et al., 2014), breast cancer classification from mammography (Kaur, 2016), and the classification of nuclear cataract severity from eye examination images (Gao et al., 2015). CNN is a biologically inspired hierarchical multilayered neural network approach that simulates the human visual cortex and detects translation invariance features (Alotaibi and Mahmood, 2016). CNN is superior to other approaches in that it conducts automatic learning for complex features from raw data and performs the classification in an end-to-end manner (Sors et al., 2018). CNN has also shown outstanding effectiveness in solving the EEG signal classification problem. Johansen et al. (2016) developed a CNN model for detecting spikes in EEGs of epileptic patients. Achilles et al. (2016) showed the superior learning performance of CNN for epileptic seizure detection. Therefore, we proposed that CNN could be used for automated detection of ripples and fast ripples in patients with intractable epilepsy. In this study, we converted a 1D intracranial EEG (iEEG) signal to 2D image signals and transformed the detection of ripples and fast ripples into a binary classification of ripples and non-ripples, as well as fast ripples and non-fast ripples. Then, a CNN model was built to classify ripples and non-ripples, as well as fast ripples and non-fast ripples. Finally, we compared the performance of our detector with the other four HFO detectors integrated in RIPPLELAB. The ultimate goal was to provide the location of EZ through the distribution of HFO generation.

## Methods

### Subjects

Patients diagnosed with medically intractable epilepsy who underwent excision of epileptic foci in the functional neurosurgery department of Xuanwu Hospital of Capital Medical University were recruited from March 2016 to May 2017. A total of 19 participants (12 males and seven females) with a mean age of 22 years (*SD* = 10; range 10–42 years) were included in the study. Intracranial data were recorded, with a sampling frequency of 4,096 Hz. Patient characteristics and electrode implantation sites are listed in Table 1. All patients gave informed consent in agreement with the Research Ethics Board of Xuanwu Hospital.

**Table 1:** Clinical characteristics and implantation sites of the 19 patients.

**Patient**	**MRI**	**Implantation sites**	**No. of channels**	**Pathology**	**Engel classification (one year after surgery)**
1	No lesion	LIF, LC, LSF, LSP, LSI, LLT	82	FCD IIb, TSC	I
2	Left DCC, left FPS, left ventricular wall ectopic	LT, LC, LP	76	FCD Ib	II
3	HS (right MTL)	LH, RH, RI	74	FCD IIIa, HS	I
4	Left temporal encephalomalacia foci	LSF, LLT, LIF, RP, RO, LO	97	HS	III
5	HS (bilateral)	RSF, RC, RIF, RSP, RLP	128	FCD I	III
6	Left temporal encephalomalacia foci	LSF, LIF, LLF, LLT, LP	96	FCD IIId	III
7	None	RLT, RP, LLT, LC, RC	112	FCD I	III
8	No lesion	RIF, RLF, RSF, RO	96	FCD I	I
9	LMS, left mastoiditis	RF, RP	80	FCD IIb	III
10	No lesion	RSF, RIF, RC, RST, RP, LIF, LSF, LC, LST, LP	96	FCD IIId	I
11	HS (left MTL)	LH, RLT, LOT	118	None	II
12	None	LOT, LIO, LP, RTH, RO, RP	96	FCD I	I
13	Abnormal signal in right cingulate gyrus	LT, RLF, RIF, RLT, RC	72	None	I
14	No lesion	RSF, RIF, RC, LSF, LIF, LC	80	FCD Ic	II
15	No lesion	RIF, RSF, RLP	62	FCD IIb	–
16	HS (right MTL)	RP, RSP, RSF, RIF	82	FCD Ia	I
17	High signal in the right frontal local cortex	RP, RPO, RIF, RLF	82	FCD Ic	I
18	HS (left MTL)	LF, LSI, LFP	90	FCD I	I
19	No lesion	RIF, RLF, LH	64	FCD IIa	I

*Gender: M, male; F, female. MRI: DCC, dysgenesis of the corpus callosum; FPS, frontoparietal schizencephaly. HS, hippocampal sclerosis; MTL, mesial temporal lobe; LMS, left maxillary sinusitis; Implantation sites: LIF, left inferior frontal; LC, left central; LSF, left superior frontal; LSP, left superior parietal; LSI, left superior insula; LLT, left lateral temporal; LT, left temporal; LP, left parietal; LH, left hippocampus; RH, right hippocampus; RI, right insula; RSF, right superior frontal; RC, right central; RIF, right inferior frontal; RSP, right superior parietal; RLP, right lateral parietal; LLF, left lateral frontal; RF, right frontal; RP, right parietal; LOT, left occipital-temporal; LIO, left inferior temporal; RTH, right temporal-hippocampus; RO, right occipital; LO, left occipital; RLF, right lateral frontal; RLT, right lateral temporal; RPO, right parietal-occipital; LF, left frontal; LFP, left frontal-cingulate. Pathologies: FCD, focal cortical dysplasia; HS, hippocampal sclerosis; TSC, tuberous sclerosis complex*.

### Data Preprocessing

We recorded interictal samples of 5 min during the slow-wave sleep period from each patient, as there is less muscle activities and more frequent occurrences of HFOs during slow-wave sleep compared with wakefulness (Zelmann et al., 2009; Burnos et al., 2014). There was also the advantage that a 5-min segment could provide the same information as a longer interval when identifying HFOs during slow-wave sleep (Zelmann et al., 2009). Slow-wave sleep was defined by at least 25% delta activity by visual inspection of 30-s epochs. Data samples were selected if they were recorded at least 2 h before or after a seizure, to reduce the influence of seizures on our analysis. Data containing noise or artifacts, such as sharp transients with very large amplitudes or irregular signals, were excluded. The data were transformed to a bipolar montage for further analysis, which means that the potential difference between two adjacent active electrodes in the skull is recorded as iEEG.

**Figure 1:**
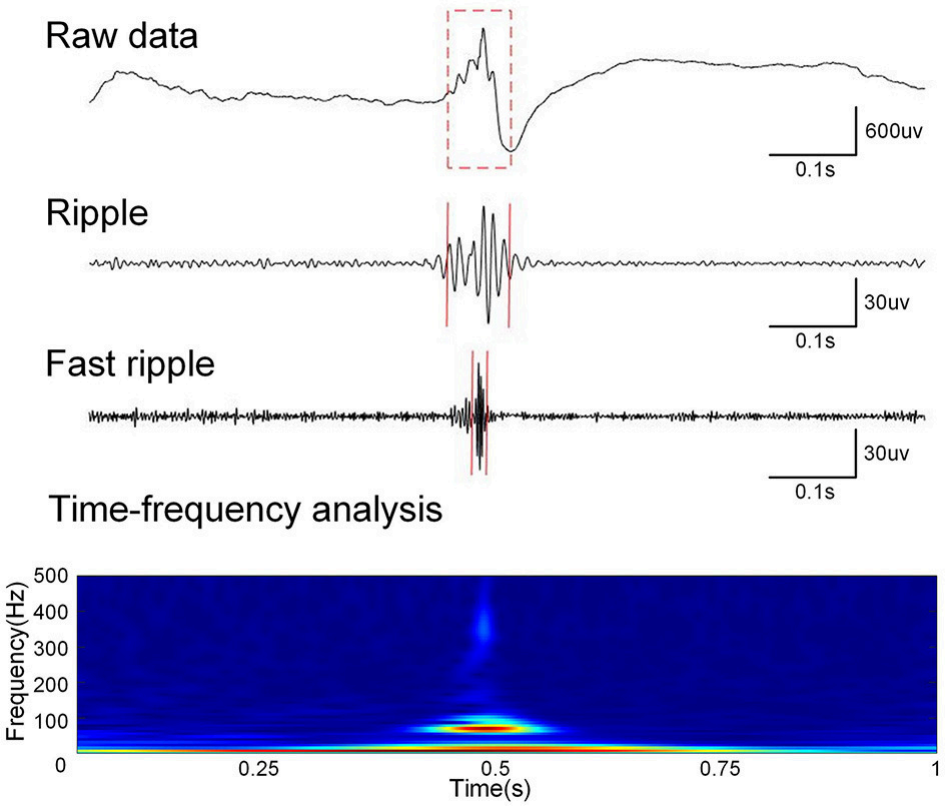
Data preprocessing. First row: one second of raw data. Second row: one second of filtered (80–200Hz) data. Third row: one second of filtered (200–500Hz) data. Fourth row: Time-frequency analysis of raw data.

The two kinds of HFOs were analyzed separately, owing to the different generation mechanisms and electrophysiological characteristics of ripples and fast ripples. A zero-phase finite impulse response filter was used to perform band-pass filtering for the data. The cutoff frequencies were 80–200 and 200–500 Hz for ripples and fast ripples, respectively (see Figure 1).

As interictal HFOs are commonly short (<330 ms) and rare (Lopez-Cuevas et al., 2013), the iEEG signals were divided into one-second time series. Grayscale was used to characterize the amplitude of iEEG signals, so that a 1D iEEG signal could be converted to a row of the 2D grayscale image (see Supplementary Figures 1, 2). Then, we converted each row of the grayscale image to four rows.

### Visual Marking of HFOs

For each channel, the first minute of the iEEG was independently analyzed by two experienced reviewers. The concordance between the two reviewers was assessed in line with the Cohen's kappa coefficient for each channel (Jacobs et al., 2010). For channels with kappa <0.5, the two reviewers worked together to review the events in the first minute and established a consensus, based on which, or if kappa >0.5, the remaining 4 min of the iEEG were marked accordingly by one of the reviewers.

Among the channels for the 19 patients, a total of 49,340 ripples and 19,734 fast ripples were analyzed by reviewers. The remaining data were tagged as non-ripples and non-fast ripples, respectively.

### CNN Classifier

**Figure 2:**
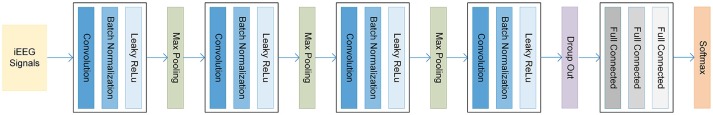
Architecture of our CNN model.

CNN requires fewer complex steps of feature extraction compared to traditional neural networks. The feature extraction is achieved by the convolutional layers and sub-sampling layers of CNN, with advantages in terms of the complex non-linear mapping of low-dimensional feature space that can be obtained from the high-dimensional feature space for use in classification. In this work, CNN has roles in both feature extraction and the classification of HFOs. Details of the proposed CNN model are shown in Figure 2.

Input images were 4^*^1,024 pixels in size, and were normalized to have zero mean and unit variance. This normalization achieves faster convergence and avoids local minima. In the model, the normalized input is processed by convolutional blocks, where each block consists of three layers: the convolutional layer, batch normalization layer and non-linear activation layer (leaky ReLU was chosen as the activation function in this study). The output of the leaky ReLU layer is passed to a max pooling layer. In an attempt to avoid overfitting, dropout is applied before the three fully connected layers. The output of the last fully connected layer is passed to a softmax layer, which serves as a classifier and predicts the class of the input signal.

#### Architecture of CNN Model

##### Convolutional layers

CNN, as a simple neural network, makes use of convolution in place of general matrix multiplication. The convolutional layers, which detect local conjunctions of features from the previous layer, constitute the main components of the CNN model. A convolutional layer consists of neurons that are connected to the local receptive field of the previous layer. The feature map of the previous layer is convoluted with the convolution kernel. Then, the activation function is applied to produce one output matrix. The process is defined as:

(1) $$\begin{array}{r} x_{j}^{l}=f\left(\sum_{i\in Mj}x_{j}^{l-1}\times k_{ij}^{l}+b_{j}^{l}\right) \end{array}$$

where *f*() represents the activation function, leaky ReLU; *l* indicates the number of layers; *k* is the kernel matrix; and *b* is a bias value.

##### Batch normalization layer

During training, the distribution of feature maps changes owing to the updating of parameters, making the CNN model learning harder to fit. This phenomenon was called covariate shift by Ioffe et al. (Ioffe and Szegedy, 2015), who proposed batch normalization as a solution. Batch normalization accelerates network training, combined with a reduction of the sensitivity to network initialization. The batch normalization layer normalizes the activations and gradients propagating through the network, making network training an easier optimization problem. In our CNN model, a batch normalization layer is applied after each convolutional layer.

##### Max pooling layer

The max pooling operation reports the maximum output within a rectangular neighborhood. This layer not only reduces the spatial size of the feature map, but also removes redundant spatial information, which is beneficial for translation and scaling of invariance to small shifts and distortions. The max pooling layer makes it possible to increase the number of filters in deeper convolutional layers without increasing the required computational load per layer.

##### Dropout layer

Dropout regularization is an effective way to address the overfitting phenomenon in the neural network training process. A dropout algorithm is applied to facilitate the generalization ability of the network by randomly disabling neurons in each layer during training.

##### Softmax layer

The softmax activation function normalizes the output of the fully connected layer. It constructs a hypothetical function to calculate the probability of the input samples being divided into each category, and then adjusts the parameters to make the correct tags corresponding to the maximum probability. The softmax activation function is deployed to approximate the expected output between 0 and 1 in our binary classification. The classification output of the network is “1” in the presence of HFOs and “0” for non-HFOs.

#### Details of Learning

After defining the network structure, we specified the training options. Our CNN model uses the minibatch and stochastic gradient descent algorithms. The minibatch is set at 256. Cross entropy serves as the loss function. The maximum number of epochs are assigned a value of 20. An epoch is a full training cycle on the entire training data set, in which the training begins with an initial learning rate of 0.01 and the learning rate decreases by a factor of five every five epochs. The CNN training was performed on an NVIDIA Quadro M4000 with computational capability of 5.9 and a clock rate of 800 MHz.

### Statistical Analysis

A 10-fold cross-validation approach, namely ten partitions for training and test sets, 90% for training and 10% for testing, was employed to measure the stability of the performance of the proposed CNN model. The performance metrics included specificity and sensitivity. Previous studies of automated HFO detection also adopted these metrics (Dumpelmann et al., 2012), and they are appropriate for comparison of our model with other methods. The calculations were as follows:

(2) $$\begin{array}{r} \text{sensitivity}=\frac{TP}{TP+FN} \end{array}$$

(3) $$\begin{array}{r} \text{specificity}=1-\frac{FP}{TP+FP} \end{array}$$

where true positive (TP) refers to the visually marked HFOs that are detected by the CNN model; false positive (FP) refers to automatically detected events that do not overlap with visually marked HFOs; and false negative (FN) means visually marked HFOs that are missed by the detector.

Cohen's kappa coefficient was computed to evaluate the agreement between automated detection and visually marked results. Kappa <0 indicates that an agreement is due purely to chance, kappa >0.5 means excellent consistency, and kappa = 1 indicates complete agreement (Zelmann et al., 2009).

Then, the Spearman's rank correlation was applied to assess the association between automated detection and visually marked results (Dumpelmann et al., 2012). The number of HFOs detected by visual marking and automated detection in each channel were counted. A correlation coefficient of 0.5–1 represented a strong correlation.

Finally, the Mann–Whitney *U*-test was applied to compare the HFO rates in the EZ channels and other channels (Dumpelmann et al., 2015).

All statistical analyses used SPSS Statistics (IBM Corporation, Armonk, NY, USA), version 22. The level of significance was set at *p* < 0.05. Results were expressed as mean ± standard deviation.

## Results

### Different Sample Sizes

Visually marked data were used to train the CNN model, consisting of HFOs and the low-amplitude activity here termed non-HFO. The ratio of HFOs to non-HFOs was 1:1. Ninety percent of the data were taken as training samples, and the model was tested on the remaining 10%. Ripples and fast ripples, representing different physiological significance, were, respectively, applied to train the CNN model.

**Figure 3:**
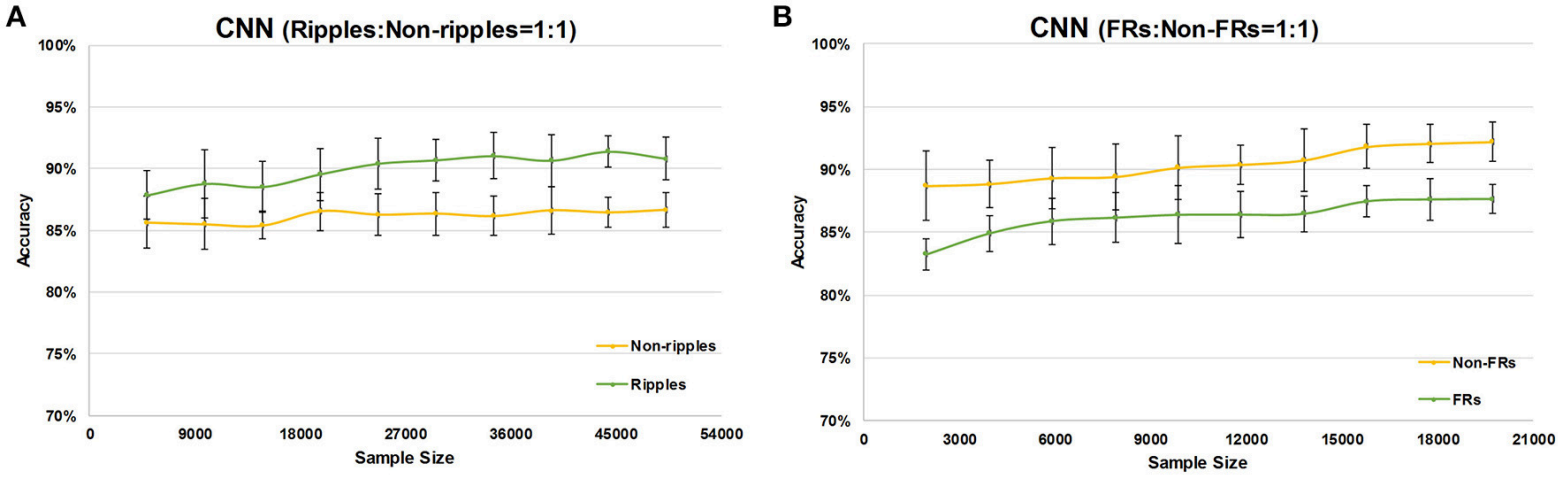
Effects of different sample sizes on CNN performance. The green line represents the accuracy of HFOs (ripples for **A** and fast ripples for **B**), and the yellow line represents the accuracy of non-HFOs (non-ripples for **A** and non-fast ripples for **B**).

We changed the number of sample data points to test whether the sample size affects CNN performance; the results are shown in Figure 3. The more training samples were used, the more accurate was the detection of HFOs. As the number of training samples increased from 4,934 to 49,340, the accuracy of ripple detection increased from 87.84+1.97 to 90.83+1.78% (see Figure 3A). Similarly, the accuracy of fast ripple detection increased from 83.25±1.27 to 87.65±1.13% as the number of training samples increased from 1,973 to 19,730 (see Figure 3B).

### Selection of the Best Model

There are numerous parameters in a CNN that have a significant impact on its classification accuracy. The settings used tend to be based on experience and practical considerations. Thus, it was important to conduct quantitative analysis of the parameters in our CNN. Seven CNN models were taken into consideration in our initial analysis to select the best model, as shown in Table 2. We performed experiments using 10-fold cross-validation with all seven models on the same sample, with a total of 48,480 ripples and 48,480 non-ripples, as well as 19,730 fast ripples and 19,730 non-fast ripples.

**Table 2:** The specifications of seven CNN models and their mean performance using 10-fold cross-validation.

**Model**		**M1**	**M2**	**M3**	**M4**	**M5**	**M6**	**M7**
Conv_1	No. of kernels	32	256	64	64	64	32	16
	Filter size	[2, 12]	[2, 12]	[2, 12]	[2, 12]	[2, 12]	[2, 12]	[2, 12]
Maxpooling_1	Pool size	[2, 4]	[2, 4]	[2, 4]	[2, 4]	[2, 4]	[2, 4]	[2, 4]
	Stride	2	2	2	2	2	2	2
Conv_2	No. of kernels	64	128	64	64	64	32	16
	Filter size	[1, 8]	[1, 8]	[1, 8]	[1, 8]	[1, 8]	[1, 8]	[1, 8]
Maxpooling_2	Pool size	[1, 4]	[1, 4]	[1, 4]	[1, 4]	[1, 4]	[1, 4]	[1, 4]
	Stride	2	2	2	2	2	2	2
Conv_3	No. of kernels	128	64	32	32	32	16	8
	Filter size	[1, 8]	[1, 8]	[1, 8]	[1, 8]	[1, 8]	[1, 8]	[1, 8]
Maxpooling_3	Pool size	[1,4]	[1,4]	[1,4]	[1,4]	[1,4]	[1, 4]	[1, 4]
	Stride	2	2	2	2	2	2	2
Conv_4	No. of kernels	256	32	32	32	32	16	8
	Filter size	[1, 8]	[1, 8]	[1, 8]	[1, 8]	[1, 8]	[1, 8]	[1, 8]
Dropout		0.5	0.5	0.5	0.5	0	0.5	0.5
Fully Connected_1		128	128	128	64	64	64	64
Fully Connected_2		64	64	64	32	32	32	32
Fully Connected_3		2	2	2	2	2	2	2
Accuracy	Ripples	92.33 ± 0.80%	90.83 ± 1.78%	92.88 ± 0.93%	93.12 ± 0.84%	92.28 ± 1.16%	92.91 ± 0.97%	92.65 ± 0.47%
	Non-ripples	87.99 ± 0.68%	86.65 ± 1.38%	87.91 ± 0.77%	88.11 ± 0.95%	88.71 ± 1.13%	87.95 ± 0.61%	87.95 ± 0.48%
	Fast ripples	87.23 ± 1.98%	87.64 ± 1.61%	88.13 ± 1.05%	87.65 ± 0.89%	87.81 ± 1.83%	88.39 ± 1.04%	88.12 ± 0.43%
	Non-fast ripples	91.63 ± 1.37%	92.21 ± 1.13%	92.34 ± 1.18%	92.82 ± 0.87%	91.64 ± 1.18%	93.35 ± 0.66%	93.28 ± 0.84%

Model M1 was designed based on the traditional concept wherein the number of kernels increases in each layer with increasing network depth, whereas in models M2 to M7 (pyramid models), the number of kernels decreased with increasing network depth. The pyramid models have the advantage of reducing the number of learning parameters compared with traditional models, which avoids the risk of overfitting.

The average performance results for 10-fold cross-validation of different models are shown in Table 2. The average accuracies (over all models) were 92.43% for ripples, 87.9% for non-ripples, 87.85% for fast ripples, and 92.47% for non-fast ripples. Based on the overall results, the pyramid models (M2 to M7) showed better performance than the traditional model (M1); in most cases, the best results were given by model M4 for ripples and M6 for fast ripples. The CNN worked better with a dropout of 0.5 and 64 neurons in the fully connected layer rather than 128 neurons. Model M4 was used to detect ripples and M6 was used for fast ripples for all further analysis in this study.

### Selection of the Ratio of HFOs to non-HFOs

**Figure 4:**
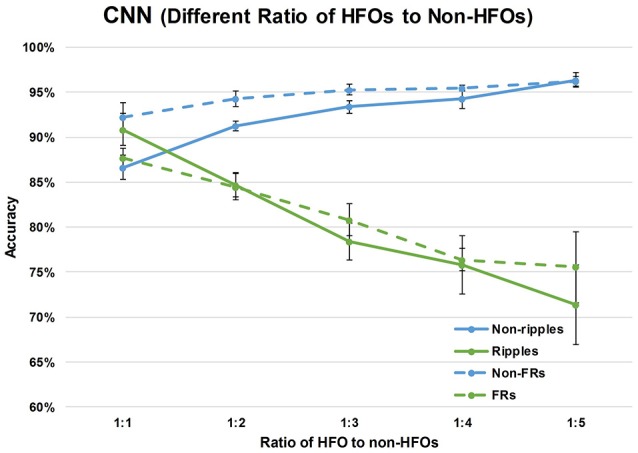
Effects of different ratios of HFOs to non-HFOs on performance of the CNN. The green solid line represents ripples, the blue solid line represents non-ripples, the green broken line represents fast ripples, and the blue broken line represents non-fast ripples.

The specificity of HFOs is correlated with the rate of false positives, that is, the automatically detected events that do not overlap with visually marked HFOs. When an HFO: non-HFO ratio of 1:1 was used to train the CNN model, the accuracy was not satisfactory with either non-ripples or non-fast ripples. In order to minimize false positive rates and improve the specificity of HFO detection, we increased the ratio of HFOs to non-HFOs by increasing the number of non-HFOs to two, three, four, and five times the number of HFOs, while keeping the number of HFOs constant. As shown in Figure 4, increasing the number of non-HFOs raised the accuracy of non-HFO detection within a certain range; on the other hand, the sensitivity of HFO detection decreased. In order to improve the specificity of HFO detection while maintaining a reasonable sensitivity, we chose the ratio of ripples to non-ripples to be 1:4, and the ratio of fast ripples to non-fast ripples to be 1:3, to train the CNN model.

### Comparison of Visual and Automated Detection Results

The CNN model based on the optimum configuration was run to test the performance objectively. In this part, data from one patient were selected as the testing samples, and data from the remaining 18 patients were selected as the training samples. The results for six patients are shown in Table 3: the average sensitivities were 77.04% for ripples and 83.23% for fast ripples, and the average specificities were 72.27% for ripples and 79.36% for fast ripples. Our automated HFO detector based on the CNN model could detect HFOs well, and there were advantages in terms of computational time. Our detector took only about 20 s to process 5 min of 90 channels iEEG data using an Intel® Xeon® CPU E5-2650 v4 @ 2.2 GHz processor and 64 GB RAM.

**Table 3:** Comparison of results between our detector and the other four detectors.

**Patient**			**1 (%)**	**2 (%)**	**3 (%)**	**4 (%)**	**5 (%)**	**6 (%)**	**Average (%)**
Our detector	Ripples	Sens	73.67	80.21	75.42	81.64	85.06	66.22	77.04
		Spec	50.47	73.15	79.59	77.15	82.25	71.03	72.27
	Fast ripples	Sens	90.66	79.55	85.50	82.40	77.73	83.54	83.23
		Spec	70.75	87.74	72.71	77.41	88.14	79.43	79.36
STE detector	Ripples	Sens	12.38	12.73	14.61	16.46	28.97	3.26	14.74
		Spec	86.21	77.71	86.86	89.29	87.32	71.43	83.14
	Fast ripples	Sens	36.00	15.79	8.54	18.81	17.66	1.44	16.37
		Spec	67.93	74.83	41.18	84.54	86.25	33.23	64.66
SLL detector	Ripples	Sens	76.23	52.00	15.94	28.57	56.27	41.48	45.08
		Spec	33.33	66.10	9.02	7.84	60.14	40.00	36.07
	Fast ripples	Sens	72.97	37.13	52.10	33.33	13.33	11.11	36.66
		Spec	58.70	74.70	66.67	45.45	30.00	3.23	46.46
HIL detector	Ripples	Sens	72.89	27.89	2.90	25.00	55.31	30.37	35.73
		Spec	54.55	83.11	25.00	46.67	89.00	85.42	63.96
	Fast ripples	Sens	12.50	19.55	22.95	51.13	26.42	22.22	25.80
		Spec	78.57	87.73	82.35	74.96	72.43	73.68	78.29
MNI detector	Ripples	Sens	26.97	8.47	31.88	28.57	9.97	0.74	17.77
		Spec	87.88	79.63	3.71	3.28	83.78	7.14	44.24
	Fast ripples	Sens	75.68	80.24	81.57	53.33	80.00	77.78	74.77
		Spec	25.45	52.91	26.72	23.53	18.56	15.38	27.09

*Sens, sensitivity; Spec, specificity*.

**Figure 5:**
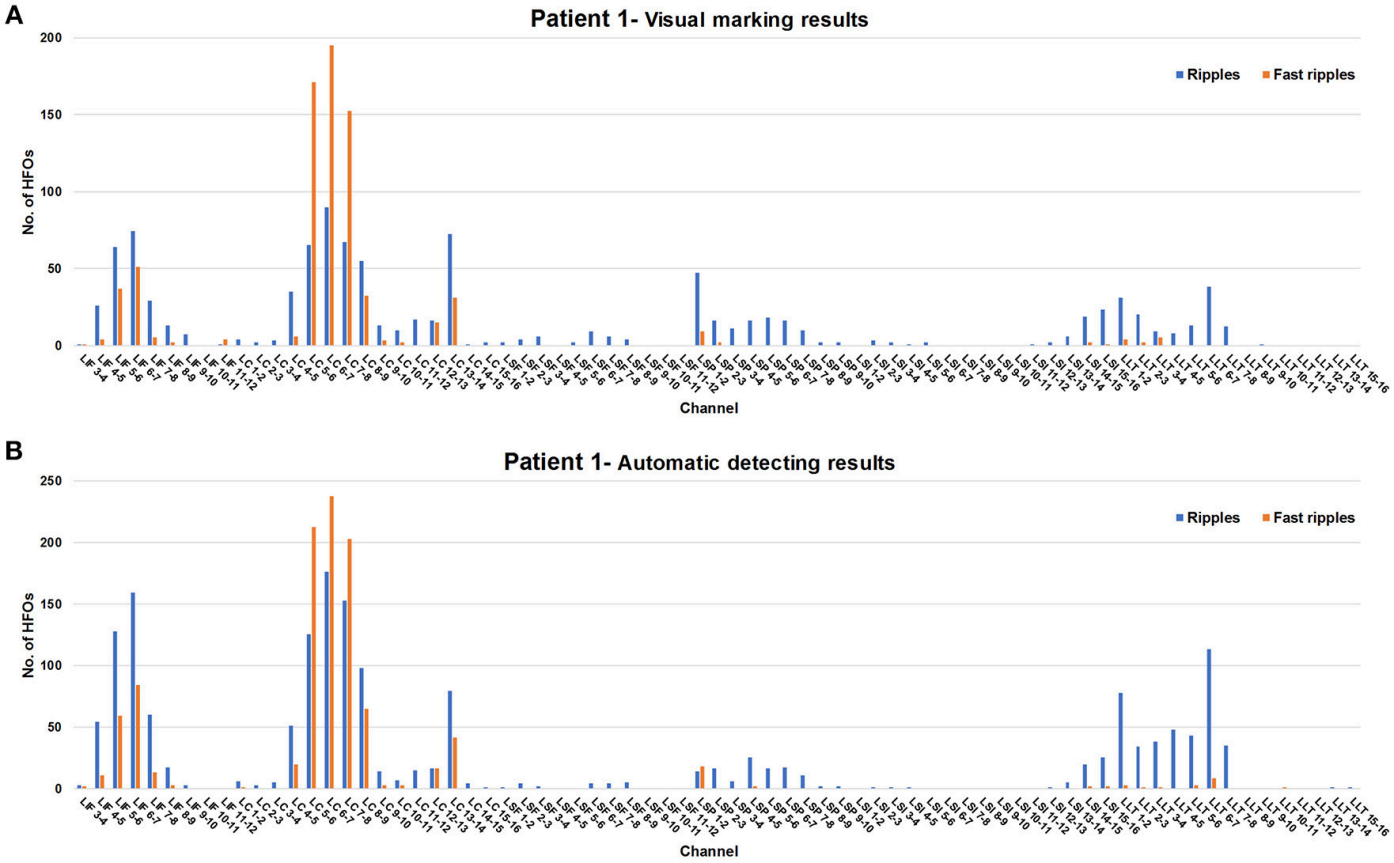
Comparison of results between visual marking and automated detection for patient 1. Blue and red represent ripples and fast ripples, respectively. **(A)** Patient 1: visual making results. **(B)** Patient 1: automatic detecting results.

At present, the most important consequence of automated detection systems is the reduction in the time required for analysis and the elimination of subjective factors. It is also necessary to ensure a strong correlation between visual and automated analysis results. In this study, we calculated the Cohen's kappa coefficient of the visual marking and automated detection results for patient 1. The kappa values for the two results were 0.541 for ripples and 0.777 for fast ripples. Spearman's rank correlation was used to calculate the correlation between the automated detection and visual analysis results for each channel. The significant correlations (0.862 for ripples and 0.938 for fast ripples, *p* < 0.01) indicated that our detector achieved reliable estimates of HFO counts and reflected the topographical distribution of HFO generation. A visual representation of the distribution of HFOs for all electrodes is displayed below in Figure 5, showing the ripple and fast ripple counts for visual analysis and automated detection for patient 1 for each channel.

### Comparison With Four Other Detectors

To evaluate the performance of our detector, it was necessary to compare its results with those of other detectors for analysis of the same data. Here, we compared our detector with four well-known detectors implemented in the RIPPLELAB application (Navarrete et al., 2016), Short Time Energy detector (STE), Short Line Length detector (SLL), Hilbert detector (HIL), and MNI detector (MNI). Detailed descriptions of this algorithm are available in the original publication (Navarrete et al., 2016). The results of our comparison are presented in Table 3. Our detector showed markedly higher sensitivity (77.04% for ripples and 83.23% for fast ripples) and specificity (72.27% for ripples 79.36% for fast ripples) than the four detectors except the specificity of STE detector for ripples (83.14%) and the sensitivity of MNI detector for fast ripples (74.77%).

### Comparison of HFO Rates in the EZ and Other Channels

In this study, we considered the brain area of the removed contacts of patient 1 as the EZ, for whom a good outcome was obtained (Engel I). The mean HFO rates in the 38 channels within the EZ were compared with those of 44 channels outside the EZ; the results are shown in Table 4. The mean HFO rates in the EZ were 32.9 for ripples and 25.4 for fast ripples. In the other channels, the mean HFO rates were 16.2 for ripples and 2.2 for fast ripples. The Mann-Whitney U-test was employed to compare the HFO rates in the EZ and other channels, showing that HFO rates were significantly higher in the EZ channels than outside (*p* < 0.05).

**Table 4:** Mean HFO rates for channels in the EZ and other channels.

**Patient 1**	**EZ channels**		**Other channels**		**Total channels**	
	**No. of HFOs**	**Mean HFO rate**	**No. of HFOs**	**Mean HFO rate**	**No. of HFOs**	**Mean HFO rate**
Ripples	1,251	32.9	712	16.2	1,963	23.9
Fast ripples	965	25.4	97	2.2	1,062	13.0

### Missed HFOs and False Detections of Our Detector

**Figure 6:**
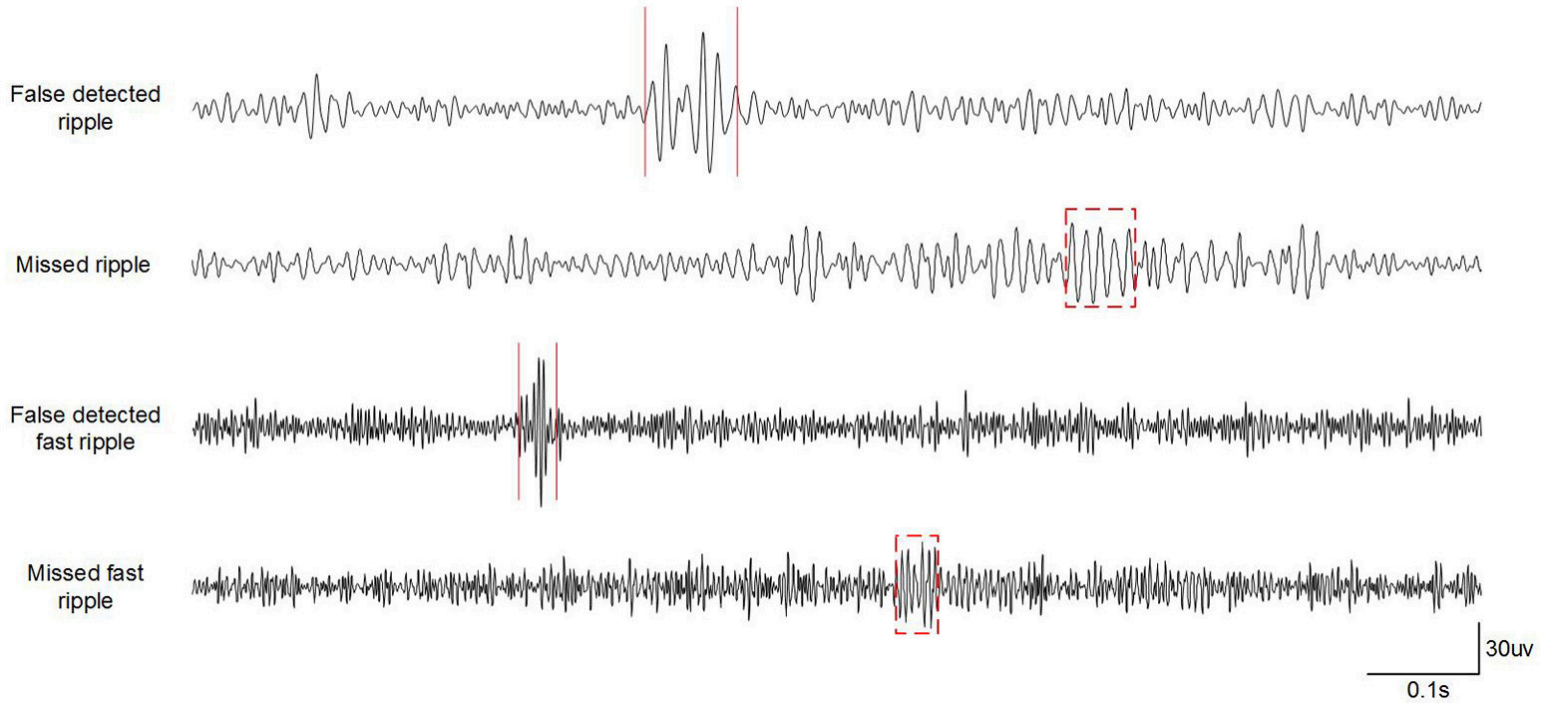
Example of missed HFOs and false detections in the first patient. The dotted rectangles represent ripples marked only by visual detections. The red lines delineate false detections.

Our detector showed excellent comprehensive performance in detecting ripples and fast ripples from iEEG signals, but there were still some missed HFOs and false detections. Some typical examples of these are shown in Figure 6. Our detector was not sensitive to HFOs with low amplitudes, and sharp transients (e.g., epileptic spikes or sharp waves) might have been misclassified as HFOs owing to their high-pass filter response as oscillations, leading to an overestimation of HFO rates (Benar et al., 2010).

## Discussion

HFOs are considered to be promising biomarkers for the identification of EZ (Jacobs et al., 2010, 2012; Cimbalnik et al., 2016). Visual marking is characterized by its heavy workload, consumption of time, and vulnerability to errors. In this study, an efficient and novel framework was integrated with CNN for the automated detection of HFOs, as a solution to this challenging medical processing problem. This approach is expected to relieve the burden on clinicians and to provide a useful tool for HFO detection in clinical settings. Compared with the four other detectors, our detector achieved better comprehensive performance: a higher sensitivity (77.04% for ripples and 83.23% for fast ripples) and specificity (72.27% for ripples and 79.36% for fast ripples). In addition, our detector could automatically analyze ripples and fast ripples separately, enabling direct comparison of HFOs in two different frequency bands. Thus, our detector has significant potential for use in clinical practice.

### Parameter Optimization

Various parameters determine both the computational performance and the accuracy of a CNN model. We compared the classification performance of our models under different parameter conditions; the results are presented in Table 2. Changes in parameters were correlated with changes in accuracy of HFOs. The models M1 and M2 achieved accuracies of 90.83 and 92.33%, respectively, for ripples, indicating that pyramid models (M2 to M7) performed better than the traditional model (M1). The early CNN model proposed by Lecun et al. (1998) introduced the strict pyramidal approach. Ullah et al. (Ullah and Petrosino, 2016) also demonstrated that giving pyramidal structure to CNNs can allow the number of parameters to be scaled down, as well as reducing memory consumption on disk; thus, the simple strict pyramidal model outperforms many existing sophisticated approaches.

As shown in Table 2, the CNN with model M4 provided the best results for ripples, while the model M6 was best for fast ripples. Both of them showed slightly higher performance than others but involved the minimum number of parameters among all the models. Model M4 and M6 were adopted for all other analysis processes in this work, as they were considered the optimal models.

### Comparison With Other Four Detectors

Several automated HFO detectors have been reported, some of which were high specific, but low sensitive. In this study, we compared our detector with the other four detectors provided by RIPPLELAB (Navarrete et al., 2016), STE detector, SLL detector, HIL detector, and MNI detector. Our detector utilized the CNN model to detect HFOs from iEEG signals. This model resulted in excellent sensitivity (77.04% for ripples and 83.23% for fast ripples) and specificity (72.27% for ripples and 79.36% for fast ripples). Our detector had a better performance than the SLL detector, HIL detector, and MNI detector. Although the STE detector had a higher specificity (83.14%) for ripples than our detector, its sensitivity (14.74% for ripples and 16.37% for fast ripples) was much lower than ours. The sensitivity is as significant as the specificity, because a detector with low sensitivity cannot delineate the distribution of HFOs in different channels, while low specificity may overestimate the amount of excitatory tissue that needs to be resected according to HFO analysis. Based on full consideration of these two factors, our detector seemed to perform better than the other four detectors. Only detectors with excellent sensitivity and specificity are appropriate for clinical use.

### Resection of HFO-Generating Areas Correlates With Outcome of Epilepsy Surgery

As was shown in many of the previous studies, brain regions with a high rate of HFOs are often correlated with EZ (Jacobs et al., 2010; Wu et al., 2010; Dumpelmann et al., 2015). Signal processing aims to detect HFOs from iEEG signals and to identify electrode sites exhibiting high HFO rates. For patient 1, the Cohen's kappa coefficient demonstrated excellent concordance between the visual marking and automated detection results (0.541 for ripples and 0.777 for fast ripples) for our detector. In addition, the high Spearman's rank correlation between the visual analysis and automated detection (0.862 for ripples and 0.938 for fast ripples, *p* < 0.01) indicated that our detector is a practical tool for identifying channels with high HFO counts. Brain areas containing LIF 3-12, LC 1-8, and LSF 1-12 were removed by surgery. As shown in Figure 5, most of the brain tissue with high HFO rates was resected, resulting in a good outcome (Engel I).

Our automated detector also provided reliable information about the distribution of HFO rates between channels (see Table 4). The mean HFO rates were significantly higher in EZ channels than elsewhere (Mann–Whitney *U*-test, *p* < 0.05). This indicates that HFO rates can provide additional information about patient outcomes.

### The Optimal Ratio of HFOs and non-HFOs

Our automated detector was designed as a supplementary diagnostic tool for the localization of EZ requiring surgical resection. Thus, the detector required good sensitivity and specificity, with a need to remove as many false positive events as possible with a reasonable sensitivity. The specificity of HFOs is correlated with the accuracy of non-HFOs. Hence, the accuracy of non-HFOs was improved so as to enhance the specificity of HFOs. When a sample with a 1:1 ratio of HFOs:non-HFOs was used to train the CNN model, the accuracy was not satisfactory for detecting either non-ripples or non-fast ripples. Subdividing the iEEG signals in HFOs and non-HFOs resulted in too many types of activities (e.g., baseline, epileptic spikes, and sharp waves) being contained in the non-HFOs, which made the non-HFO data insufficient. Therefore, we increased the number of non-HFOs to two, three, four, and five times the number of HFOs, with the number of HFOs kept constant, so as to improve the accuracy of non-HFOs. As shown in Figure 3, increasing the number of non-HFOs did raise the accuracy of non-HFOs within a certain range, on the other hand, the sensitivity of HFOs decreased. To improve the specificity of HFOs with a reasonable sensitivity, we chose a ratio of ripples to non-ripples of 1:4, and a ratio of fast ripples to non-fast ripples of 1:3, to train the CNN model.

### Limitations and Future Work

Although the CNN model overcame some important issues in HFO detection, there still were some limitations. A potential weakness of implementing the CNN model in this way is that it did not utilize any cross-channel information. Moreover, the CNN model could not obtain the start and stop time, amplitude, or energy of HFOs. Future work should focus on further enhancement of performance of the CNN model.

## Conclusion

With the continuous accumulation of medical data, there is an increasing need for the feature extraction and classification to predict class labels for patient's clinical data. In this study, we present an efficient detector powered by the CNN to detect ripples and fast ripples automatically. This method has achieved satisfactory performance compared with existing approaches, which might be utilized in a clinical setting in the future. Our detector is, therefore, valuable for identifying EZ during pre-surgical or intraoperative evaluation.

## Author Contributions

RZ and JW designed the study, implemented the algorithms, performed data analysis, and wrote the manuscript. XL, CZ, and CJ selected and pre-processed the iEEG data. YL and CL performed data analysis and helped to produce the tables and figures. XG and ZR provided advice and guidance. XY and XZ provided the research ideas and revised the manuscript. All authors revised and approved the final version of the article.

### Conflict of Interest Statement

The authors declare that the research was conducted in the absence of any commercial or financial relationships that could be construed as a potential conflict of interest.
