# Supplementary material for: Automated Detection of High-Frequency Oscillations in Epilepsy Based on a Convolutional Neural Network
Source: Front Comput Neurosci. 2019 Feb 12;13:6. doi: 10.3389/fncom.2019.00006 (PMC6379273; doi:10.3389/fncom.2019.00006)
Supplement: Supplementary file 1 [file Table_1.docx]

Supplementary Material

Automated Detection of High-Frequency Oscillations in Epilepsy Based on a Convolutional Neural Network

Rui Zuo,^1,2†^ Jing Wei,^1,2†^ Xiaonan Li,^3,4^ Chunlin Li,^1,2^ Cui Zhao,^1,2^ Zhaohui Ren,^1,2^ Ying Liang,^1,2^ Xinling Geng,^1,2^ Chenxi Jiang,^3,4^ Xiaofeng Yang,^3,4^* and Xu Zhang^1,2^*

*** Correspondence:**

Dr. Xiaofeng Yang

[xiaofengyang@yahoo.com](mailto:xiaofengyang@yahoo.com)

Dr. Xu Zhang

[zhangxu@ccmu.edu.cn](mailto:zhangxu@ccmu.edu.cn)

# Supplementary Figure


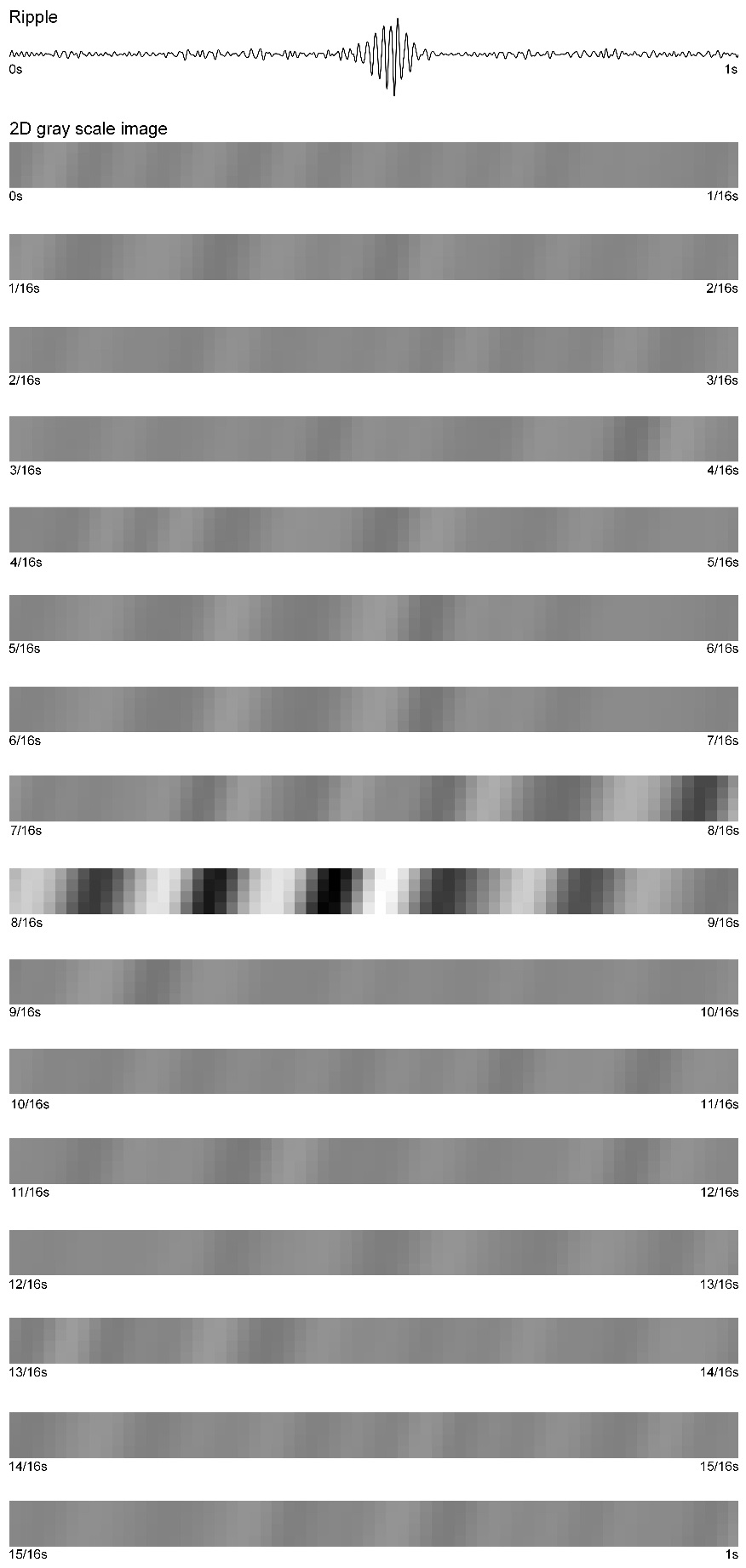


**Supplementary Figure 1.** First row: one second of filtered (80–200 Hz) data. A four-row grayscale image of one second is displayed on the second to last row.


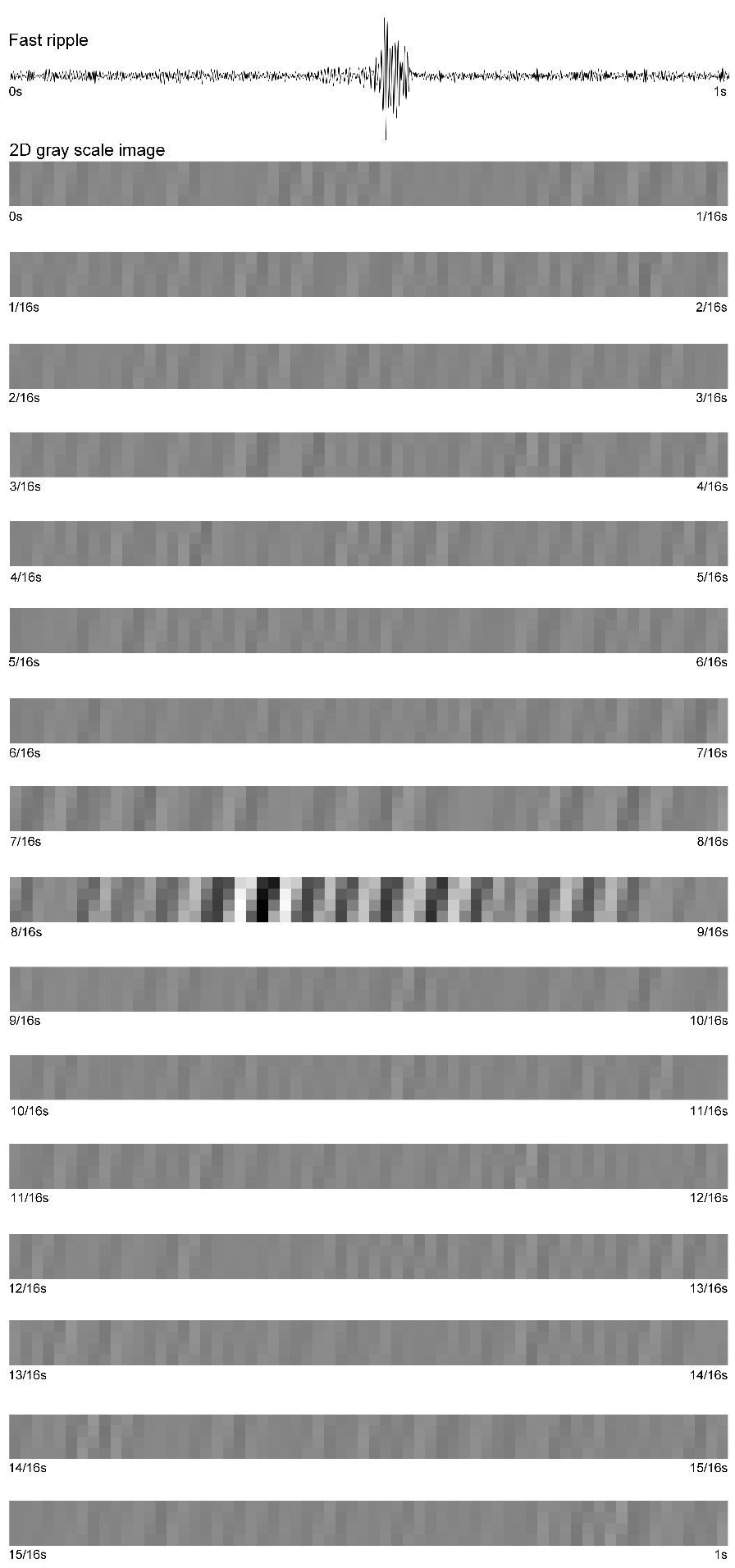


**Supplementary Figure 2.** First row: one second of filtered (200–500 Hz) data. A four-row grayscale image of one second is displayed on the second to last row.
